# Parsing Age Effects in Future Self-Continuity through Goal Orientation and Life Satisfaction

**DOI:** 10.1093/geroni/igaf122.3015

**Published:** 2025-12-31

**Authors:** Laken Mooney, Ryan Best

**Affiliations:** West Virginia University, Morgantown, West Virginia, United States; West Virginia University, Morgantown, West Virginia, United States

## Abstract

Future self-continuity (FSC), the feeling of connectedness between the current and the future self, has been shown to predict positive outcomes such as dieting and the likelihood of saving for retirement. Limited additional work has investigated the trajectory of FSC across adulthood, generally finding that FSC increases with age. Possible mechanisms considered in this work such as future time perspective, cognition, personality, and health were not found to account for the observed age effects in multiple regression models. The current study builds on this earlier work by using a parallel mediation model to test if age related differences in goal orientation and life satisfaction may account for age-related increases in FSC. An online sample of American adult participants (n = 479) was collected from Prolific. Using a hierarchical regression model, previous findings were replicated showing age as a significant predictor of FSC after controlling for income, education, time perspective, goal orientation, and life satisfaction (p < .001). Using a parallel mediation model, we found significant indirect effects for gain-oriented goal orientation (β = .022) and life satisfaction (β = .016) but not for maintenance or loss prevention goal orientation. Increased age was associated with a decrease in gain orientation which in turn is associated with a decrease in FSC while age was associated with an increase in life satisfaction which was associated with an increase in FSC. Results are discussed in light of the role of our goals and satisfaction in life in establishing a sense of continuity.

